# High Levels of Cartilage Oligomeric Matrix Protein in the Serum of Breast Cancer Patients Can Serve as an Independent Prognostic Marker

**DOI:** 10.3389/fonc.2019.01141

**Published:** 2019-10-30

**Authors:** Konstantinos S. Papadakos, Amélie Darlix, William Jacot, Anna M. Blom

**Affiliations:** ^1^Division of Medical Protein Chemistry, Department of Translational Medicine, Lund University, Malmö, Sweden; ^2^Department of Medical Oncology, Institut Régional du Cancer Montpellier ICM, University of Montpellier, Montpellier, France

**Keywords:** COMP, ELISA, prognostic marker, taxanes, Docetaxel, Paclitaxel

## Abstract

**Background:** Cartilage oligomeric matrix protein (COMP) is a pentameric cartilage protein also expressed in breast cancer tumors. A high expression of COMP evaluated by immunohistochemical staining is as an independent prognostic marker associated with poor patients' prognosis.

**Methods:** Herein, levels of COMP were analyzed using an IVD approved ELISA in serum samples from 233 well-characterized breast cancer patients; 176 with metastatic breast cancer; and 57 in an early stage of the disease.

**Results:** The metastatic patients had double the concentration of serum COMP compared with those with early breast cancer. High levels of COMP in sera of metastatic patients were associated with the histological subtype (*p* = 0.025) and estrogen receptor positivity (*p* = 0.019) at the time of breast cancer diagnosis. Further, correlation was observed between the serum levels of COMP and the presence of liver (*p* = 0.010) or bone (*p* = 0.010) metastases in this population. Most importantly, elevated serum levels of COMP appear to serve as an independent prognostic marker of survival as assessed by Cox proportional hazard regression analysis (*p* = 0.001) for the metastatic patients. Among metastatic patients treated with taxanes (Docetaxel-Paclitaxel) as part of their first metastatic line (*n* = 25), those with high levels of serum COMP detected in the metastatic stage of the disease had a shorter median survival (0.2 years) compared with those with low levels of serum COMP (1.1 years) (*p* = 0.001).

**Conclusions:** Taken together, the serum levels of COMP are elevated in the metastatic patients and may be a potential novel biomarker for the evaluation of the prognosis in this population.

## Introduction

Cartilage oligomeric matrix protein (COMP), abundant in cartilage, was unexpectedly found to be expressed in tumor tissues from breast (1), prostate (2), and colon cancer (3). A strong COMP expression in tumor cells was recently correlated with reduced breast cancer-specific survival and recurrence-free survival of breast cancer patients as an independent prognostic marker (1). Moreover, COMP expression in tumor tissues correlated positively with the presence of lymph nodes metastases, and estrogen/progesterone receptor positivity (1).

COMP belongs to the thrombospondin proteins family (4), alternatively named TSP5, with each molecule comprising 5 monomers bound together at the N-terminus of the protein, forming a pentamer. COMP was first described as an extracellular matrix protein that plays a crucial role in the organization of cartilage (5). Accordingly, mutations in the COMP gene can lead to pseudoachondroplasia and multiple epiphyseal dysplasia (6). COMP is also highly expressed in fibrotic scars, particularly in scleroderma (7, 8). In diseases that lead to the destruction of cartilage, such as osteoarthritis, the elevated levels of COMP in serum serve as an independent prognostic marker for cartilage turnover (9, 10). Moreover, COMP contributes to the maintenance of vascular homeostasis since COMP degradation by ADAMTS-7 regulates vascular remodeling, and COMP has been found in atherosclerotic plaques and lesions contributing to restenosis of the artery (11). Furthermore, it has been revealed that COMP regulates coagulation through the inhibition of thrombin (12).

One molecular mechanism by which COMP leads to the poor survival of breast cancer patients has recently been revealed (13). COMP expression leads to a larger cancer stem cell population *in vitro* and in *vivo*, as a result of Notch3 pathway activation. Notch3 is activated when it binds to its ligand Jagged1, and COMP can bind both molecules (Notch3 and Jagged1) and increase their interaction, leading to a higher activation of the Notch pathway and cross-talking with other important cancer related molecular pathways, such as AKT and β-catenin (14). Previously, it has been shown that COMP expressing cells are resistant to apoptosis induction, endoplasmatic reticulum stress, and upregulate the Warburg effect (1, 2). Some of these effects of COMP can be ascribed to its ability to disrupt Ca^2+^ signaling.

Levels of COMP in serum can be measured using an IVD (*in vitro* diagnostics) approved ELISA, with a reported cut-off of 12 U/L for the evaluation of aggressive joint destruction (15). In breast cancer, COMP expression has been evaluated in tumor tissue samples by immunostaining, but to date no study has evaluated the prognostic value of COMP serum levels. In this study we aimed to determine whether patients with metastatic cancer have higher levels of serum COMP than patients with early breast cancer, correlate the serum levels of COMP in advanced breast cancer patients with the pathophysiological characteristics of tumors, and to evaluate if COMP serum levels could also be used as an independent prognostic marker as described using immunochemical staining in tumor tissue samples (1). Serum measurements of COMP could allow a much easier evaluation compared with the more invasive immunohistochemical analysis of patient tumor tissues.

## Materials and Methods

### Cohort Description

Breast cancer patients were retrospectively identified by reviewing the medical records of the breast cancer patients from the Montpellier Cancer Institute database between 2008 and 2015. Inclusion criteria were: patient ≥ 18 years old; histologically confirmed breast cancer; availability of the hormone receptor (HR) and HER2 statuses of the primary tumor; availability of a frozen serum sample performed at the early or metastatic phase, for biomarker determination. Serum samples from the early breast cancer patients were obtained before surgical removal of the primary tumor and without neoadjuvant treatment or any indication of metastasis. For the metastatic breast cancer patients, the serum samples were acquired after at least one confirmed metastasis, with a median time of 15 months since the date of first metastasis and a range of 123 months. Patients with history of other cancer(s) were excluded. Clinical and biological data were collected by reviewing the medical records of the selected patients: demographical, clinical (date of diagnosis of breast cancer and, if applicable, metastatic disease; metastatic status at breast cancer diagnosis; treatment history including number of metastatic treatments to take into account the variable sampling time in this population), and biological data (histological grade of the primary tumor, HR and HER2 statuses). The tumor was considered HR-positive when more than 10% of cells were labeled in immunohistochemistry or when the concentrations of estrogen (ER) and progesterone receptors (PR) using the radio ligand binding method were above 10 and 50 ng/mL, respectively. The tumor was considered HER2-positive if the primary tumor was scored 3+ by immunohistochemistry or if the HER2 gene was amplified by fluorescence or chromogenic *in situ* hybridization (FISH/CISH) for immunohistochemistry 2+ cases. For cases with HR and/or HER2 status changes over time, the status used was that of the most recent sample. For cases of synchronous or asynchronous bilateral cancer with discrepant HR and/or HER statuses, the most unfavorable biology was used: higher histological grade, HR-negative, HER2-negative (Trastuzumab era). None of the selected patients had an inflammatory joint disease (rheumatoid arthritis, ankylosing spondylitis or other chronic inflammatory diseases of the joint requiring a specific treatment).

Primary tumor tissue blocks of a subpopulation of patients selected for the availability of serum samples were selected from the Biological Resource Center of the Montpellier Cancer Institute (Biobank number BB-0033-00059) for immunohistochemical COMP evaluation. The study was reviewed and approved by the Montpellier Cancer Institute Institutional Review Board (ID number CM-CORT- 2018-04).

### Objectives

The primary objective of the study was to determine whether patients with metastatic breast cancer have higher levels of serum COMP than patients with early breast cancer, and if the serum COMP levels correlate with the severity of the disease in the metastatic patients. The secondary goal was to evaluate if COMP serum level can serve as independent prognostic marker for survival of the metastatic patients as was previously observed for immunohistochemical analysis of COMP in tumor tissues. Sample size for comparison of metastatic and early breast cancer patients was calculated with bilateral α risk = 5%, β = 20% (power = 80%), difference in COMP serum level (Δ) = or > 3 U/L (hypothesis: mean level in the early breast cancer group = or <12 U/L), standard deviation = 12. Under such assumptions, the numbers of patients to be included were at least 224 of which 56 in the early breast cancer group and 168 in the metastatic group.

### COMP Serum Levels: ELISA Method

The COMP serum levels of patients were measured using an IVD approved ELISA method (AnaMar AB) following manufacturer instructions. In brief, serum was diluted (1/10) in the provided sample buffer (20 μl serum +180 μl sample buffer), added to precoated 96-well plate, together with enzyme conjugated antibody and incubated for 2 h at room temperature. The plates were then washed 6 times, developed with TMB substrate and measured at 450 nm using Cytation 5 (Biotek). Results were calculated with Prism 8 statistical analysis software (GraphPad) using Cubic Spline regression. Every patient serum was measured in duplicate, and samples outside calibration curve were further diluted and remeasured.

### COMP Tumor Tissue Expression: Immunohistochemical Staining

Breast cancer tissue was mounted using FLEX system microscope coated slides. Antigen retrieval was performed with Envision Flex high pH kit (Dako) using a PT-link module (Dako). Tissues were stained with 0.47 μg/ml rabbit polyclonal affinity purified anti-COMP in-house antibody previously evaluated for its specificity (1), utilizing Envision Flex (Dako) reagents in the Autostainer Plus system according to the manufacturer's protocol (Dako). Slides were scanned with Aperio Scanner system (Leica) at 40X and intensity of COMP evaluated in a blinded fashion using scores: 0 for negative staining, 1 for low expression, 2 for moderate expression and 3 for high expression.

### Statistical Analyses

Sample distribution was assessed with Kolmogorov-Smirnov and Shapiro-Wilk normality tests. Spearman's rank correlation coefficient was used to demonstrate correlations between scale variables and Mann-Whitney or Kruskal-Wallis for the rest of the variable combinations as indicated in each table. The overall survival (OS) was calculated from the date of the serum sampling until death or last news (data collected until 20th March 2019), assessed applying the Kaplan-Meier method and displayed as medians and survival rates with their 95% confidence intervals. Prognostic values of COMP serum levels were evaluated using hazards ratios, with Cox proportional hazards regression model with a stepwise procedure and bootstrap replications. Statistical significance was considered when two side *p*-values were smaller than 0.05. All calculations were performed with the SPSS statistical analysis software version 24 (IBM).

## Results

### Patients Characteristics

In this study we included in total 233 women of which 176 (75.5%) had metastatic breast cancer and 57 (24.5%) early breast cancer, with a median age of 55 years (range: 27–87 years) (Table 1). We did not observe any statistically significant difference between the two groups as far as the median age at the time of the serum sampling, histological subtype, histological grade (SBR), estrogen receptor (ER) status, and progesterone receptor (PR) status. As expected, variations could be detected for parameters that characterize the metastatic risk and the stage of the disease as well as human epidermal growth factor receptor 2 (HER2) status (*p* = 0.017), distant metastases, type of surgery, axillary node dissection, radiotherapy, number of previous systemic therapy lines, adjuvant treatment, neoadjuvant treatment (*p* < 0.001) and tumor size (*p* = 0.014) (Table 1).

**Table 1:** Patients characteristics in the metastatic and the early breast cancer groups.

**Factor**	**Patients *N* (%)**	**Metastatic BC N (%)**	**Early BC N (%)**	***p-*value**
All	233 (100.0)	176 (75.5)	57 (24.5)	
Age *at the time of the serum sample*	233 (100.0)	176 (75.5)	57 (24.5)	0.055
<50	93 (39.9)	65 (27.9)	28 (12.0)	
50–70	105 (45.1)	81 (34.8)	24 (10.3)	
>70	35 (15.0)	30 (12.9)	5 (2.1)	
Previous metastatic therapy (*N*) *at the time of the serum sample*	233 (100.0)	176 (75.5)	Not applicable	** <0.001**
0–2	168 (72.1)	111 (47.6)		
3–8	65 (27.9)	65 (27.9)		
Histological Subtype *at the time of BC diagnosis*	223 (100.0)	168 (75.3)	55 (24.7)	0.298
Ductal	201 (90.1)	149 (66.8)	52 (23.3)	
Lobular	16 (7.2)	13 (7.7)	3 (1.3)	
Mixed	6 (2.7)	6 (3.6)	0 (0.0)	
Missing	10 (4.3)	8 (3.4)	5 (2.14)	
Histological grade (SBR) *at the time of BC diagnosis*	216 (92.7)	159 (73.1)	57 (26.9)	0.236
1–2	94 (43.5)	73 (33.8)	21 (9.7)	
3	122 (56.5)	86 (39.8)	36 (16.7)	
Missing	17 (7.3)	17 (7.3)	0 (0.0)	
ER status *at the time of BC diagnosis*	233 (100.0)	176 (75.5)	57 (24.5)	0.983
Negative	127 (54.5)	96 (41.2)	31 (13.3)	
Positive	106 (45.5)	80 (34.3)	26 (11.2)	
PR status *at the time of BC diagnosis*	233 (100.0)	176 (75.5)	57 (24.5)	0.211
Negative	174 (74.7)	135 (57.9)	39 (16.7)	
Positive	59 (25.3)	41 (17.6)	18 (7.7)	
HER2 status *at the time of BC diagnosis*	232 (99.6)	175 (75.4)	57 (24.6)	**0.017**
Negative	123 (53.0)	85 (36.6)	38 (16.4)	
Positive	109 (47.0)	90 (38.8)	19 (8.2)	
Missing	1 (0.4)	1 (0.4)	0 (0.0)	
Distant metastases *at the time of BC diagnosis*	233 (100.0)	176 (75.5)	57 (24.5)	** <0.001**
M0	166 (71.2)	109 (46.8)	57 (24.5)	
M1	55 (23.6)	55 (23.6)	0 (0.0)	
Mx	12 (5.2)	12 (5.2)	0 (0.0)	
Surgery *at the time of BC diagnosis*	233 (100.0)	176 (75.5)	57 (24.5)	** <0.001**
No	30 (12.9)	30 (12.9)	0 (0.0)	
Tumorectomy	112 (48.1)	73 (31.3)	39 (16.7)	
Mastectomy	91 (39.1)	73 (31.3)	18 (7.7)	
Axillary node dissection *at the time of BC diagnosis*	219 (94.0)	162 (74.0)	57 (26.0)	** <0.001**
Yes	39 (17.8)	38 (17.4)	1 (0.5)	
No	180 (82.2)	124 (56.6)	56 (25.6)	
Missing	14 (6.0)	14 (6.0)	0 (0.0)	
Tumor size *at the time of BC diagnosis*	207 (88.8)	151 (72.9)	56 (27.1)	**0.014**
≤2 cm	51 (23.7)	44 (21.3)	7 (3.4)	
>2 cm	156 (76.3)	107 (51.7)	49 (23.7)	
Missing	26 (11.2)	25 (10.8)	1 (0.4)	
Radiotherapy *at the time of BC diagnosis*	233 (100.0)	176 (75.5)	57 (24.5)	** <0.001**
No	54 (23.2)	52 (22.3)	2 (0.9)	
Breast	36 (15.5)	25 (10.7)	11 (4.7)	
Breast-lymph nodes	143 (61.4)	99 (42.5)	44 (18.9)	
Adjuvant treatment *at the time of BC diagnosis*	233 (100.0)	176 (75.5)	57 (24.5)	** <0.001**
None	125 (53.6)	93 (39.9)	32 (13.7)	
Hormonotherapy	55 (23.6)	31 (13.3)	24 (10.3)	
chemotherapy	21 (9.0)	21 (9.0)	0 (0.0)	
Hormone-chemotherapy	32 (13.7)	31 (13.3)	1 (0.4)	
Neoadjuvant treatment *at the time of BC diagnosis*	233 (100.0)	176 (75.5)	57 (24.5)	** <0.001**
No treatment	125 (53.6)	125 (53.6)	0 (0.0)	
Treatment	108 (46.4)	51 (21.9)	57 (24.5)	

*BC, Breast cancer; COMP, cartilage oligomeric matrix protein; ER, estrogen receptor; HER2, human epidermal growth factor receptor 2; PR, progesterone receptor; SBR, Scarff-Bloom-Richardson. Pearson's χ^2^ test two-tailed p-value. The bold indicates p < 0.05*.

### Comparison of COMP Immunohistochemical Staining With COMP Serum Levels (*n* = 39)

**Figure 1:**
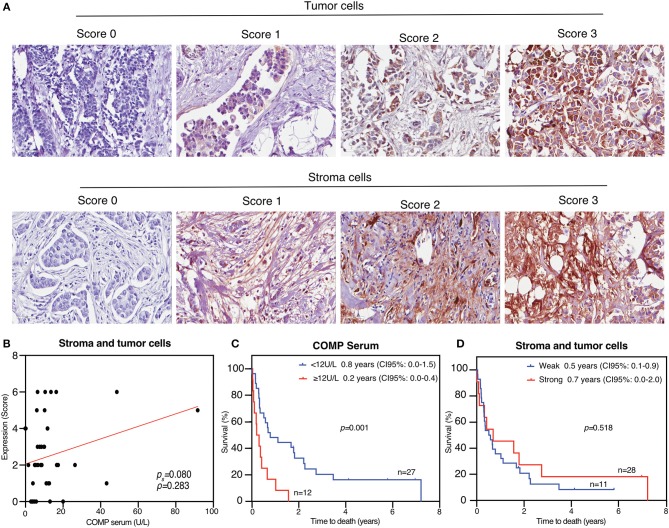
COMP is expressed in breast cancer and can be measured in patient sera, which correlates with poor survival. **(A)** Biopsies from 39 metastatic breast cancer patients were stained for COMP and the intensity of the staining in tumor cells and stroma was scored separately. Representative pictures for each score were taken at 40X magnification. **(B)** The scores were grouped into weak (scores 0–2) and strong (score 3) COMP expression. Correlation between level of COMP in sera and cumulative score for immunohistochemical signal in stroma and tumor cells applying the Spearman rank correlation. **(C)** High serum COMP (≥12 U/L) are correlated with shorter overall survival (OS). **(D)** Strong COMP expression in stroma and tumor cells was not significantly associated with decreased OS. Kaplan-Meier analyses with Breslow's test was used in **(C,D)**, *p* < 0.05 were considered significant and survival is presented as the median.

In the first stage of this study, 39 patients with metastatic breast cancer were included (median age 57 years, range: 33–84 years) with one later excluded due to an unsuccessful immunohistochemical stain. Tumor biopsy samples obtained from the primary tumor (for 34 patients) or metastatic tissue (for 5 patients) were immunohistochemically stained for COMP. The intensity of expression was scored (Figure 1A). In agreement with the previous report (1), COMP expression was found at a varying intensity in both the stroma and the tumor cells. COMP levels were then measured in sera of the same patients using ELISA (note that serum samples were not collected at the same time as biopsies). Normality test for the distribution of the obtained values revealed that these do not follow the normal distribution; as a consequence, non-parametric statistical tests were applied. A tendency for significant correlation was detected between the tissue expression of COMP (both from the tumors cells and stroma cells) and the serum levels of COMP (*p* = 0.080) (Figure 1B). For the assessment of cartilage turnover and the risk of joint destruction the patients are categorized in the following risk groups: <12 U/L COMP as low risk of aggressive joint destruction and value found in a healthy population, 12–15 U/L as increasing risk of aggressive joint destruction and ≥15 U/L as high risk of aggressive joint destruction (10 U/l is equal to ~1 μg/ml of serum COMP) (16). Thus, we divided our initial patient samples in two categories: <12 U/L as negative samples for the presence of COMP in serum and ≥12 U/L as positive samples for the presence of COMP in serum. Using the 12 U/L cut-off, we observed that patients with COMP positive sera (≥12 U/L) had lower median OS (median OS: 0.2 years, 95% CI: 0.0–0.4) compared with those that had low serum levels of COMP (<12 U/L) (median OS: 0.8 years, 95% CI: 0.0–1.5, *p* = 0.001), according to Kaplan-Mayer survival analysis (Figure 1C). In contrast, the COMP immunohistochemical stains in this relatively small group of metastatic patients (*n* = 39) failed to show any difference (*p* = 0.518) in median OS between patients that had a strong (score 3) expression of COMP in tumor or stroma cells (median OS: 0.7 years, 95% CI: 0.0–2.0) and those who had a weak expression (score 0–2) (median OS: 0.5 years, 95% CI: 0.1–0.9; Figure 1D). A larger study, specifically designed to find the optimal cut off value for COMP may identify even more accurate cut-off value for metastatic status in breast cancer.

### Correlation Between Serum Levels of COMP and Clinical Characteristics (*n* = 233)

Based on the promising data from the first stage of the study, and considering also the fact that the tissue and serum samples were not collected at the same time (primary tumor tissue in most cases and serum sample collected at the metastatic phase of the disease in all cases) we included further 193 patients, for a total of 233 patients with early and metastatic breast cancer and a median age of 55 years (range: 27–87 years), and measured COMP levels in sera (Table 2).

**Table 2:** Association between COMP serum levels and clinical characteristics of the metastatic patients.

**Factor**	**Patients *N* (%)**	**COMP ELISA (U/L)**	***p-*value**
All	176 (100)	Mean (SD)	
Age *at the time of the serum sample*	176 (100)		0.643^a^
<50	65 (36.9)	11.32 (12.52)	
50–70	81 (46.0)	11.65 (4.57)	
>70	30 (17.0)	31.13 (39.41)	
Histological subtype *at the time of BC diagnosis*	168 (95.5)		**0.025^b^**
Ductal	149 (88.7)	10.30 (10.23)	
Lobular-Mixed	19 (11.3)	17.80 (23.06)	
Missing	8 (4.5)		
Histological grade (SBR) *at the time of BC diagnosis*	159 (90.3)		0.097^b^
1–2	73 (45.9)	11.69 (11.93)	
3	56 (54.1)	10.86 (13.54)	
Missing	17 (9.7)		
ER status *at the time of BC diagnosis*	176 (100)		**0.019^b^**
Negative	96 (54.5)	9.69 (10.50)	
Positive	80 (45.5)	12.36 (14.01)	
PR status *at the time of BC diagnosis*	176 (100)		0.398^b^
Negative	135 (76.7)	10.84 (12.44)	
Positive	41 (23.3)	11.11 (11.78)	
HER2 status *at the time of BC diagnosis*	175 (99.4)		0.585^b^
Negative	85 (48.6)	12.23 (15.20)	
Positive	90 (51.4)	9.73 (8.59)	
Missing	1 (0.6)		
Adjuvant treatment *at the time of BC diagnosis*	176 (100)		0.062^a^
None	93 (52.8)	8.75 (8.13)	
Hormonotherapy	31 (17.6)	15.33 (18.40)	
chemotherapy	31 (17.6)	13.02 (16.67)	
Hormone-chemotherapy	21 (11.9)	10.80 (5.26)	
Distant metastasis *at the time of BC diagnosis*	176 (100)		**0.014**^**a**^
M0	109 (61.9)	12.05 (13.63)	
M1	55 (31.3)	7.67 (4.82)	
Mx	12 (6.8)	15.36 (19.12)	
Bone metastasis *at the time of the serum sample*	176 (100)		**0.010^b^**
Absence	79 (44.9)	9.33 (10.64)	
Presence	97 (55.1)	12.19 (13.35)	
Liver metastasis *at the time of the serum sample*	176 (100)		**0.010^b^**
Absence	72 (40.9)	8.35 (9.01)	
Presence	104 (59.1)	12.67 (13.84)	
Brain metastasis *at the time of the serum sample*	176 (100)		0.53^b^
Absence	121 (68.8)	10.09 (8.38)	
Presence	55 (31.3)	12.68 (18.06)	
Lung metastasis *at the time of the serum sample*	176 (100)		0.558^b^
Absence	88 (50.0)	10.70 (10.68)	
Presence	88 (50.0)	11.10 (13.72)	
Subcutaneous metastasis *at the time of the serum sample*	176 (100)		0.368^b^
Absence	151 (58.8)	10.43 (10.40)	
Presence	25 (14.2)	13.77 (20.26)	
Metastasis of other sites *at the time of the serum sample*	176 (100)		0.658^b^
Absence	122 (69.3)	10.62 (11.95)	
Presence	54 (30.7)	11.54 (13.03)	
Previous therapy (N) *at the time of the serum sample*	176 (100)		0.370^b^
0–2	111 (63.1)	10.02 (10.61)	
3–8	65 (36.9)	12.42 (14.62)	

*COMP, cartilage oligomeric matrix protein; ER, estrogen receptor; HER2, human epidermal growth factor receptor 2; PR, progesterone receptor; SBR, Scarff-Bloom-Richardson*.

a *Kruskal-Wallis Test two-tailed p-value*,

b *Mann-Whitney two-tailed p-value. The bold indicates p < 0.05*.

A normality test for the distribution of the sample revealed that it does not follow the normal distribution, and non-parametric statistic tests were therefore applied. Within the metastatic patient population, the average levels of serum COMP (10.30 U/L) were significantly lower in patients with ductal subtype (*n* = 149) compared with the lobular and mixed histological subtype (*n* = 19, average levels 17.80 U/L; Table 2). ER positive tumors were associated (*p* = 0.019) with higher average serum COMP levels (*n* = 80, 12.36 U/L) compared with ER negative tumors (*n* = 96, 9.69 U/L), in accordance with previous observations in 2 breast cancer patients cohorts (1). Also, tumors with 1 or 2 SBR grade (*n* = 93) were associated (*p* = 0.035) with marginally higher levels (10.00 U/L) of COMP in serum compared with those with SBR grade 3 (*n* = 123, 9.03 U/L). The HER2, PR status, the age, the SBR histological grade and type of adjuvant treatment were not correlated with the levels of serum COMP (Table 2).

### Elevated Levels of Serum COMP in Patients With Metastatic Breast Cancer (*n* = 176)

**Figure 2:**
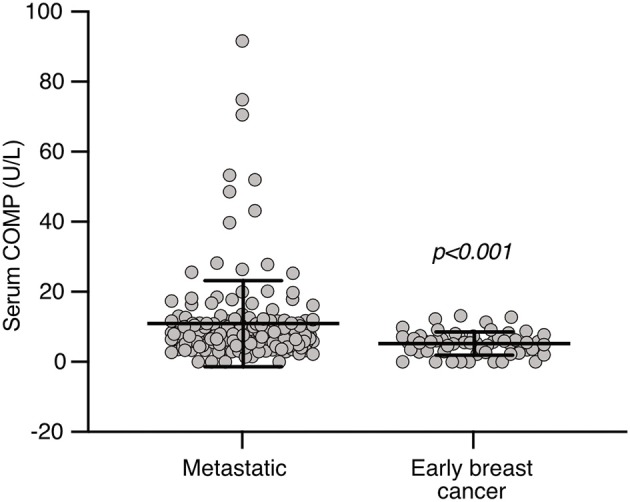
COMP levels in patient sera. COMP levels were measured in sera using IVD approved ELISA and significantly higher levels were detected in sera of patients with metastatic breast cancer compared to early breast cancer. Mann-Whitney two-tailed *p*-value.

The mean level of serum COMP of the metastatic patients (*n* = 176) was 10.72 U/L, twice as high compared to 5.22 U/L of the patients with early breast cancer (*n* = 57), with very strong statistical significance (*p* < 0.001, Figure 2). Furthermore, in the group of metastatic patients, those with bone metastases at the time of the serum sample (*n* = 97) had elevated levels (12.19 U/L) of serum COMP in comparison with those that had metastases to other sites (9.33 U/L, *n* = 79, *p* = 0.010). An association (*p* = 0.010) was also observed for 104 patients with liver metastases and higher levels of serum COMP (12.67 U/L compared with 8.35 U/L in 72 patients without liver metastases) (Table 2). The presence of metastases to the brain, lung, subcutaneous, or any other analyzed sites was not associated with the serum levels of COMP (Table 2).

### COMP Serum Levels Can Serve as an Independent Prognostic Marker for Survival in Patients With Metastatic Breast Cancer (*n* = 176)

**Figure 3:**
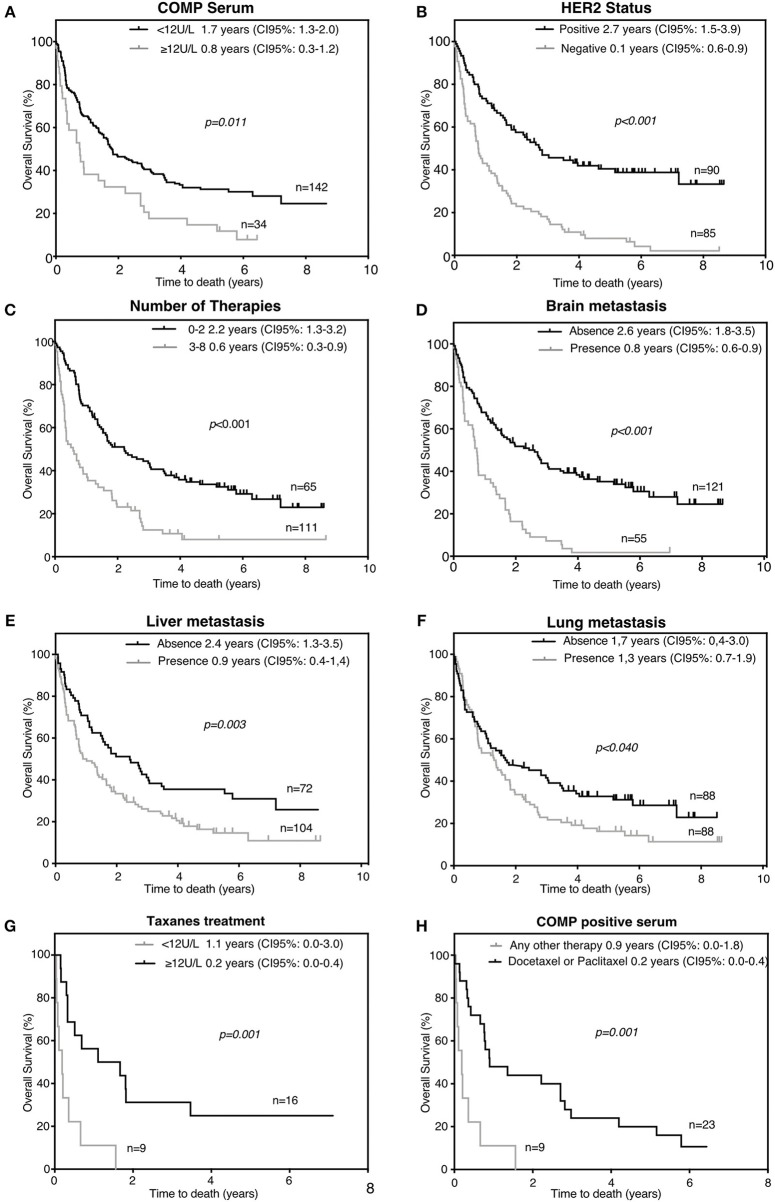
Factors associated with decreased overall survival (OS) of the metastatic patients (*n* = 176). High serum COMP levels **(A)**, HER2 negativity **(B)**, number of previous therapies **(C)**, presence of brain **(D)**, liver **(E)**, or lung **(F)** metastases were correlated with lower OS. **(G)** High serum COMP levels are associated with worse OS in patients treated with taxanes. **(H)** Breast cancer metastatic patients with positive serum COMP ELISA had poorer OS when taxanes was used as part of their first line therapy. Kaplan-Meier analysis with Breslow's test was used, *p* < 0.05 were considered significant, and survival is presented as the median.

For further calculations, we dichotomized the metastatic population of patients in two categories: COMP serum negative (*n* = 142) with serum levels <12 U/L and COMP serum positive (*n* = 34) with serum levels ≥12 U/L. In univariate analysis, the presence of COMP in the serum was strongly (*p* = 0.011) associated with a shorter OS according to Kaplan-Meier test, with a median OS of 0.8 years (95% CI: 0.3–1.2) compared to 1.7 years (95% CI: 1.3–2.0) in those with negative serum COMP (Figure 3A). Furthermore, as expected, HER2 negative tumors (with median OS 0.1 years, 95% CI: 0.6–0.9) and higher numbers of previous therapies (with median OS 0.6 years, 95% CI: 0.3–0.9) were associated with worse prognosis according to Kaplan-Meier (*p* < 0.001) estimation of survival (Figures 3B,C). The presence of metastases in brain (median OS = 0.8 years, 95% CI: 0.6–0.9), liver (median OS = 0.9 years, 95% CI: 0.4–1.4), or lungs (median OS = 1.3 years, 95% CI: 0.7–1.9) were associated with worse prognosis for patients (Figures 3D–F).

In patients with metastatic breast cancer, high levels of serum COMP were closely correlated (*p* = 0.001) with worse prognosis and could serve as an independent prognostic marker in multivariable Cox analysis regarding OS (hazard ratio, HR = 2.20). In the same multivariate analysis, the HER2 status (HR = 0.29, 95% CI: 0.19–0.45, *p* < 0.001) and the number of previous metastatic therapies (HR = 2.29, 95% CI: 1.49–3.54, *p* < 0.001) were also associated with survival (Table 3). In accordance, elevated serum COMP levels (≥12 U/L) were independently associated to OS (*p* = 0.012) with HR 1.67 (95% CI: 1.12–2.50), in Cox univariable analysis. In the same analysis HER2 status (HR = 0.36, 95% CI: 0.25–0.51) and the number of previous therapies (HR = 2.38, 95% CI: 1.68–3.37) (Table 3), were also prognostic markers.

**Table 3:** Cox univariable and multivariable survival analyses in the metastatic breast cancer patient population.

**Overall Survival**	**Univariable**			**Multivariable**		
**Variable**	**HR**	**95% CI**	***p*-value**	**HR**	**95% CI**	***p*-value**
Serum COMP levels (<12 U/L vs. ≥12 U/L)	1.67	1.12–2.50	**0.012**	2.20	1.36–3.56	**0.001**
Histological grade (SBR)	1.13	0.79–1.62	0.498	1.19	0.78–1.81	0.414
Histological Subtype	0.94	0.54–1.63	0.819	0.10	0.48–2.06	0.998
ER	0.91	0.65–1.27	0.572	0.95	0.56–1.61	0.848
PR	0.87	0.58–1.30	0.487	0.60	0.32–1.13	0.116
HER2	0.36	0.25–0.51	** <0.001**	0.29	0.19–0.45	** <0.001**
Tumor size (≤ 2 cm vs. >2 cm)	1.22	0.81–1.84	0.350	1.34	0.84–2.13	0.215
Distant metastases at BC diagnosis	0.88	0.66–1.17	0.383	1.16	0.81–1.66	0.415
Number of metastatic therapies (0–2 vs. 3–8)	2.38	1.68–3.37	** <0.001**	2.29	1.49–3.54	** <0.001**

*COMP, cartilage oligomeric matrix protein; ER, estrogen receptor; HER2, human epidermal growth factor receptor 2; PR, progesterone receptor; SBR, Scarff-Bloom-Richardson. The bold indicates p < 0.05*.

### Presence of COMP in Metastatic Breast Cancer Patient Serum Associates With Taxanes as First Systemic Therapy (*n* = 176)

In a previous study we showed that prostate cancer cells that express COMP *in vitro* were resistant in Docetaxel induced apoptosis (2). In the current study in the metastatic population of patients in total 25 patients received taxanes as adjuvant first systemic therapy (Docetaxel or Paclitaxel). Of these patients, those with high COMP (*n* = 9) serum levels at the time of serum sample (≥12 U/L) had median OS survival 0.2 years (95% CI: 0.0–0.4) compared with those (*n* = 16) with negative COMP serum levels (<12 U/L) with median OS survival 1.1 years (95% CI: 0.0–3.0), according to Kaplan-Meier (*p* = 0.001) estimation of survival (Figure 3G). Correspondingly, in the group of COMP serum positive patients from the metastatic population (*n* = 32) we compared the survival of those (*n* = 9) who received taxanes as first systemic therapy for metastatic disease (Docetaxel or Paclitaxel) with patients (*n* = 23) who received any other kind of treatment. Patients who received taxanes had a shorter survival (median OS = 0.2 years CI: 0.0–0.4), compared with those who receive any other kind of therapy (OS = 0.9 years 95% CI: 0.0–1.8), according to Kaplan-Meier (*p* = 0.001) estimation of survival (Figure 3H).

## Discussion

Initially, the expression of COMP by breast cancer tumor cells has been associated with poorer prognosis of patients (1). In recent studies, COMP has also been associated with worse outcome when it is expressed in prostate cancer (2), in colon cancer (3, 17) as well as in hepatocellular carcinoma (18). In all of these studies, COMP was found to be an independent prognostic marker by Cox multivariate analysis. The method of choice was the evaluation of COMP expression by immunohistochemical staining of patients biopsies for prostate cancer and colon cancer; in the case of hepatocellular carcinoma an ELISA was used that is not IVD approved (18). Immunohistochemical evaluation of COMP expression in tumor tissue is a diagnostic method that has several limitations, as it is both semi-quantitative and invasive, especially for the testing of metastatic tissue. For these reasons, the purpose of this study was to evaluate a new non-invasive way for the detection of COMP expression in tumors, utilizing an IVD approved ELISA that can evaluate the levels of COMP in sera of cancer patients.

In a small initial patient cohort analyzed in this study, the expression of COMP by immunohistochemical staining failed to predict breast cancer patients' survival. In contrast, a positive ELISA COMP serum sample (≥12 U/L) was able to predict worse survival for a breast cancer patient in the same population of patients. This indicates that measurement of serum COMP levels may be a more sensitive prognostic marker than immunohistochemical staining of tumor samples. One possible explanation for this observation may be that in order to yield detectable levels of COMP in serum, the breast tumors must locally express high quantities of COMP, which is then strongly correlated with late stage disease and worse prognosis (1). Additionally, one must take into consideration that ELISA as a method is more sensitive, and also quantitative compared with the immunohistochemical tissue staining. However, it must be also considered that this observation may be influenced by the difference in time of sampling as the tissue samples were collected earlier than the sera.

The mechanisms behind the observation that COMP expression in cancer is detrimental may rest in the fact that COMP expressing breast cancer tumors have a larger proportion of cancer stem cells, as described previously (13). Several reports in the literature had revealed a connection between cancer stem cells and initiation of metastasis (19), suggesting that those are the ones that can not only attach in the distant site forming metastasis, but also can self-renew and give rise to all the different cells that are encountered in a tumor (20, 21). Accordingly, in our study, the mean levels of serum COMP were closely correlated with the presence of metastases in the bone and in the liver. COMP is present on the tumor cell surface and it is also known to interact with several components of cartilage and bone. Therefore, metastatic cancer cells that express COMP may be attaching more strongly to the bone tissue (22), fit better to the local microenvironment of the tissue and thus proliferate and survive better in this niche. Indeed, under physiological conditions COMP plays a crucial role in the organization of cartilage (23) and bone development supported by the fact that mutations in COMP are associated with pseudoachondroplasia and multiple epiphyseal dysplasia (6). The association between COMP expression and presence of liver metastases may be perhaps related to the fact that COMP participates in the pathogenesis of other liver related diseases, such as liver fibrosis (24) and can serve as biomarker of liver fibrosis in patients with chronic viral hepatitis (25, 26).

We showed previously that COMP is expressed in prostate cancer where it is correlated with a poorer survival of patients. Moreover, we showed that COMP expressing prostate cancer cells develop resistance to apoptosis triggered by different compounds with exception of those that target the nuclear topoisomerase (2). One of the tested compounds that COMP expressing cells exhibited resistance against was Docetaxel, which is broadly used as first line chemotherapy drug in breast cancer (27). Accordingly, in this study we found that metastatic patients with high levels of serum COMP in the later stages of the disease treated with taxanes adjuvant in their first systemic treatment (Docetaxel or Paclitaxel), had much worse prognosis compared with patients with high levels of serum COMP but treated with any other kind of therapy. One limitation of this study to be considered is the fact that serum samples in the metastatic population were collected after the taxanes treatment, which was given in the early stages of the disease. However, one may hypothesize that the worse prognosis of serum COMP positive patients under the treatment of taxanes is due to COMP-mediated apoptotic resistance by a similar calcium metabolism related mechanism as described in prostate cancer (2). The aforementioned result emerges from a small sample of patients but could be a promising opportunity to identify patients unlikely to respond to taxanes treatment, allowing a tailoring of their first-line treatment. However, a validation in a larger patient cohort in which serum samples collected at the time of taxanes treatment are analyzed for COMP presence remains mandatory before drawing any firm conclusions.

## Conclusion

Taken together, our results show that elevated levels of COMP in sera can be detected in metastatic breast cancer patients compared with those in early stages of the disease. Additionally, serum COMP can serve as an independent prognostic marker for metastatic patients. In this population, serum COMP levels were strongly associated with a poorer prognosis in the multivariate Cox proportional hazard regression analysis. Thus, COMP levels in sera can be measured using a commercial, IVD approved ELISA method, which yields results that can be immediately applied in oncological clinics for breast cancer patient stratification and therapeutic tailoring.

## Data Availability Statement

The datasets generated for this study are available on request to the corresponding author.

## Ethics Statement

The studies involving human participants were reviewed and approved by Montpellier Cancer Institute Institutional Review Board. The patients/participants provided their written informed consent to participate in this study.

## Author Contributions

KP analyzed and interpreted the patient data and wrote the manuscript. AD collected the samples and contributed to writing of the manuscript. WJ contributed in the design of the study and was a contributor in writing the manuscript. AB designed and supervised the study and contributed to writing of the manuscript.

### Conflict of Interest

The authors declare that the research was conducted in the absence of any commercial or financial relationships that could be construed as a potential conflict of interest.

## Abbreviations

COMP: cartilage oligomeric matrix protein
OS: overall survival
ELISA: Enzyme-Linked Immunosorbent Assay
IVD: *in vitro* diagnostics
CI: confidence intervals
HR: hazard ratio
ER: estrogen receptor
HER2: human epidermal growth factor receptor 2
PR: progesterone receptor
SBR: Scarff-Bloom-Richardson.
